# Treatment of Cholera Infantum

**Published:** 1866

**Authors:** 


Treatment of Cholera Infantum.—Prof. Pollitzer, of Vienna, in a treatise on this subject, published in the *Oesterr. Jahrb. fur Kinderheilk*, strongly recommends the use of camphor in acute intestinal catarrh and cholera infantum—the earlier in the disease the better.

